# Symptom burden and follow-up of patients with neck and back complaints in specialized outpatient care: a national register study

**DOI:** 10.1038/s41598-024-53879-3

**Published:** 2024-02-15

**Authors:** John Bjørneboe, Andrea Bratsberg, Jens Ivar Brox, Sigrid Skatteboe, Maja Wilhelmsen, Kjetil Magne Samuelsen, Gunn Hege Marchand, Tonje Flørenes, Maja Garnaas Kielland, Cecilie Røe

**Affiliations:** 1 Department of Physical Medicine and Rehabilitation, Oslo University Hospital, Oslo, Norway; 2 Oslo Centre for Biostatistics and Epidemiology, University of Oslo, Oslo, Norway; 3 Department of Physical Medicine and Rehabilitation, University Hospital of North Norway, Tromsø, Norway; 4 Norwegian Neck and Back Registry, UNN Tromsø, Tromsø, Norway; 5 Department of Physical Medicine and Rehabilitation, St Olavs Hospital, Trondheim University Hospital, Trondheim, Norway; 6 Department of Physical Medicine and Rehabilitation, Haukeland University Hospital, Bergen, Norway; 7 Department of Health Care Coordination, Health Literacy and Health Equity, Oslo University Hospital, Oslo, Norway; 8 Medical Faculty, Institute of Clinical Medicine, University in Oslo, Oslo, Norway

**Keywords:** Neck pain, Back pain, Register-based study, Patient reported outcome measure, Treatment, Health care, Musculoskeletal system

## Abstract

Back and neck pain are common in the population, especially among immigrants. In Norway's specialist care system, treating these patients typically involves a multidisciplinary approach based on the biopsychosocial model. However, language and cultural differences may create barriers to participation. Immigrants are often underrepresented in clinical studies, but a register-based approach can enhance their participation in research. This study aimed to compare both the symptom burden, and treatment, among Norwegians, non-Norwegians, and patients requiring translator service for back and neck pain within the Norwegian specialist care system. The Norwegian neck and back registry is a National Quality Register, established in 2012 and fully digitized in late 2020. The baseline data includes demographics and patient recorded outcome measures including Oswestry Disability Index, Fear-Avoidance Beliefs, pain rating on a numeric rating scale, Hopkins Symptom Checklist and EuroQol five-dimensional questionnaire on health related quality of life. During the two-year study period, a total of 14,124 patients were invited, and 10,060 (71%) participated. Norwegian patients reported less pain, better function assessed by Oswestry Disability Index, lower fear avoidance beliefs, less emotional distress, and higher health related quality of life compared to non-Norwegians. We found that patients with female gender, who were younger, more educated and exhibited fear-avoidance behavior were significantly more likely to receive multidisciplinary treatment. We found no difference in the proportion of Norwegian and non-Norwegian patients receiving multidisciplinary treatment [odds ratio (OR) 1.02 (95% confidence interval (CI) 0.90–1.16)]. However, patients needing a translator were less likely to receive multidisciplinary treatment compared to those who didn't require translation [OR 0.41 (95% CI (0.25–0.66)]. We found that non-Norwegian patients experience a higher symptom burden compared to Norwegian. We found that both non-Norwegians and patient in need of translator were to a greater extent recommended treatment in primary health care. The proportion of non-Norwegians patients receiving multidisciplinary treatment was similar to Norwegians, but those needing a translator were less likely to receive such treatment.

## Introduction

Back and neck pain are the leading causes of disability globally^1^. Previous studies^2^ have found that the prevalence of musculoskeletal disorders (MSD) varies considerably throughout Europe, potentially caused by cultural differences in health-seeking behavior, socioeconomically status and working status. In Norway, one study^3^ found that 18% of men and 27% of females report MSD lasting for more than six months, with the pain most frequently localized to the low back and neck area. In a study among immigrants, Kjøllesdal^4^ found that immigrants had a higher prevalence of MSD, including elevated rates of both back and neck pain in comparison to non-immigrants in Norway.

Psychosocial factors are known to be associated with chronic neck and back pain. Previous studies^5–7^ have found that negative beliefs about back pain, emotional distress and fear-avoidance beliefs are associated with persistent back pain. Individuals reporting back and neck pain have also showed worse self-reported health status^8^.

Reports from Norway^9^ have found that immigrants have worse living conditions compared to native Norwegians, but there are large individual variations between different immigrant groups^9^. Over the last decade, several studies have evaluated both the frequency of back and neck pain and different treatment modalities. Most of these studies^10–12^ tend to have low immigrant representation. There seems to be a knowledge gap concerning immigrant patients with back and neck pain, yet they represent a considerable part of the patient population in Norwegian outpatient clinics and may have higher disability and treatment needs. From both an ethical and legal standpoint, it is crucial that patients have equal rights and equal access to treatment within the healthcare system.

Register based scientific approaches^13^ have the potential to reduce selection bias and are suitable to enhance immigrant participation in research. In Norway, a national register exists for patients with back and neck pain referred to specialized care, the Norwegian Neck and Back Register (NNRR). This register includes demographic information, pain, disability, and health status. All specialized outpatient clinics in physical medicine and rehabilitation examining patients and offering non-operative treatment in Norway are obliged to deliver data.

Thus, the aim of this study is to assess the prevalence of persons with immigrant background in the Norwegian Neck and Back Register; compare their symptoms and health to non-immigrants, and finally evaluate the multidisciplinary treatment offered to non-Norwegians in the Norwegian specialist care system.

## Methods and patients

### Design

The current study is a register-based cohort study with data from the Norwegian neck and back registry (NNRR) in 2021 and 2022. NNRR is a National Quality Register that was established in 2012 and fully digitized in late 2020. The register collect data from the baseline consultation in the outpatient unit and after 6- and 12-months follow-up. For this study we only include data from the baseline consultation.

### Participants and procedures

Patients with back and neck pain are invited to participate in the registry 16 days prior to their scheduled appointment in the specialist health care service. The participation is voluntary, and patients are included after signing an informed written consent form. The consent form and patient reported outcome measures are in both Norwegian and English. After the primary appointment in the specialist health care system the health worker responsible for the patient will fill out the consultant form (physician or physiotherapist).

### Patient reported information and outcomes

Demographic information in the patient questionnaire includes age, gender, nationality (Norwegian, European, Asian, African, American), need for interpretation support, education and work status and literacy. In the current study, we will refer to people as non-Norwegian if they have marked their nationality as European, Asian, African or American. Based on the need for translator service, the participants are either classified as in need of translator or no-translator.

Self-reported health status includes pain location, previous treatment (physiotherapy guided exercise treatments, psychomotor physiotherapy treatment, chiropractic or manual therapy treatment, surgery and medication), sick leave and workability, patient opinion on cause of injury (home, work leisure related, malpractice, muscle, skeletal or nerve related). The patient questionnaire includes several patient recorded outcome measures (PROM). Pain intensity during activity and rest during the past week was reported on an 11-point numeric rating scale (NRS)^14^, ranging from 0 (lowest pain) to 10 (highest pain). The neck disability index (NDI) and Oswestry disability index (ODI)^15–19^ consists of 10 items, ranging from 0–5. In ODI and NDI the summed score is presented as a percentage, where 0% indicates no pain related disability and 100% maximum pain related disability^15–19^.

Hopkins Symptom Checklist 10 (HSCL-10)^20–22^ measures the emotional distress experienced over the last 14 days. The 10 items are scored from 1 (not at all) to 4 (very much), and the mean is calculated and reported. A cut-off value of ≥ 1.85 for the average score is considered a valid for predicting mental distress^21^. Fear avoidance is measured through Waddell Fear Avoidance Belief Questionnaire (FABQ)^23,24^ and scored from 0 (strongly agree) to 7 (strongly disagree), where high scores indicate fear avoidance beliefs. FABQ-work consists of 7 items, and ranges from 0 to 42. FABQ-activity consists of 4 items and ranges from 0 to 24. The prevalence of health complaints is measured through the subjective health complaint inventory (SHC)^25^. All health complaints are graded from 0 (no complaint) to 3 (severe) over the last month. Finally, health related quality of life for the last 30 days is measured through EuroQol five-dimensional questionnaire (EQ-5D-5L)^26–28^. The five dimensions included in the questionnaire are Mobility, Self-Care, Usual activity, Pain/Discomfort, and Anxiety/Depression and each domain has 5 levels from no problem (1) to extreme problem (5). EQ-5D is reported on a scale from 0 (a state as bad as being dead) and 1 (full health), with a cut-off value of ≥ 0.75 considered as normal health related quality of life.

### Medical information and health services

The health professionals provide information regarding previous surgical interventions, the medical diagnosis (ICD-10), use of medication, results of radiological assessments. The follow-up plan for the patient is registered. The follow-up plan is either referral to surgery, individual and group based multidisciplinary treatment in the specialist health care system, non-multidisciplinary follow-up in the specialist health care system, recommendations for follow up in primary care (general practitioner, physiotherapist, psychologist, chiropractor), or to the Norwegian Labor and Welfare Service (NAV) or institution based rehabilitation). Multidisciplinary treatment involves more than one of the following groups: physician, physiotherapist, psychologists, occupational therapists and social workers. The multidisciplinary follow-up offered in the outpatient clinics can be either individual or group based.

### Analyses and statistics

The statistical analysis was performed in R using the standard "stats” library. We present descriptive data as means (standard deviations). Welch’s two sample t-test was used to compare Pain (NRS), ODI, HSCL-10, FABQ, UNI, EQ5D5L between Norwegian/Non-Norwegian and Translator/No translator. For comparing the groups Norwegian/Non-Norwegian and Translator/No translator with respect to treatment type (multidisciplinary treatment or not), Fisher’s exact test was performed. We also fitted separate linear regression models with pain (NRS), ODI, HSCL-10, FABQ, UNI, EQ5D5L as outcome variables, and controlling for age, gender, work status and education (categorized in three levels: vocational studies, higher education and primary and secondary school as reference level) in each model. We performed a logistic regression analysis to compare the groups Norwegian and Non-Norwegian with respect to treatment type, while controlling for education, age, gender, work status (working vs. non-working), and ODI, FABQ-total, NRS activity and EQ-5D-5L.

### Ethics

The Ministry of Health and Care services approved the Norwegian neck and back registry as a national medical quality registry in 2011. A national expert group annually reviews the quality. Participation is based on written consent. This study was approved by the Data Protection Officer (22/07399) at Oslo University Hospital.

### Guideline statement

All the methods were performed in accordance with relevant guidelines and regulations.

## Results

In 2021 and 2022, a total of 14,124 patients were referred to Physical Medicine and Rehabilitation departments with back and neck pain and participating in the NNRR. The NNRR includes 10,060 of these (71%) with both patient form and consultant form. Of these, 301 forms did not include information regarding nationality, thus excluded from the analysis (Table 1). All forms included information regarding translator (Table 1).

**Table 1:** Patient characteristics from the Norwegian neck and back registry (number and %).

Sex (n = 10,060)	
Female	5444 (54%)
Male	4616 (46%)
Age (n = 10,060)	
18–49 years	5931 (59%)
50–67 years	3315 (33%)
67 + years	814 (8%)
Education (n = 9686)	
Primary and secondary school	2441 (25%)
Vocational studies	3246 (33%)
University < 4 years	2011 (21%)
University ≥ 4 years	1988 (20%)
Nationality (n = 9759)	
Norway	8431 (86%)
Europe	753 (8%)
Africa	78 (0.8%)
Asia	307 (3%)
Central and North America	30 (0.3%)
South America	18 (0.2%)
Other	142 (2%)
Translator (n = 10,060)	
No	9911 (98.5%)
Yes	149 (1.5%)

Patients with Norwegian nationality reported less pain on NRS in both activity and rest, have better function assessed by ODI and have less fear-avoidance belief in both activity and work, less emotional distress, less subjective health complaint and higher health related quality of life compared to non-Norwegians (Table 2). These differences were also significant when adjusting for age, gender, work status and education (Table 2).

**Table 2:** Comparison of means (standard deviation) for background data and PROMs for Norwegians and non-Norwegians.

	Norwegian (n = 8431)	Non-Norwegian (n = 1328)	Mean difference (95% CI)	Adjusted difference
Age	46.7 (14.9)	42.9 (10.6)		
Female	55%	48%		
Working (%)	48%	44%		
NRS activity	6.5 (2.2)	7.3 (2.2)	0.8 (0.7, 0.9)	0.8 (0.7, 0.9)
NRS rest	5.4 (2.3)	6.1 (2.3)	0.7 (0.6, 0.9)	0.7 (0.6, 0.8)
ODI	31.5 (14.9)	37.8 (16.5)	6.2 (5.3, 7.2)	6.1 (5.2, 6.9)
FABQ activity	13.4 (5.3)	16.3 (5.6)	2.9 (2.6, 3.2)	2.7 (2.4, 3.0)
FABQ work	21.1 (11.2)	26.4 (11.4)	5.3 (4.6, 6.0)	4.6 (4.0, 5.2)
HSCL 10	2.0 (0.6)	2.2 (0.7)	0.14 (0.10, 0.18)	0.12 (0.08, 0.15)
UHI	19.8 (11.0)	22.3 (12.8)	2.5 (1.8, 3.2)	2.4 (1.8, 3.0)
EQ5D5L	0.66 (0.22)	0.61 (0.24)	− 0.05 (− 0.06, − 0.03)	− 0.04 (− 0.05, − 0.03)

Mean difference with 95% Confidence interval (CI). The adjusted difference is calculated through linear regression, with age, gender, education and work status as covariates. Numeric rating scale (NRS) ranges from 0 to 10, Oswestry Disability Index (ODI) from 0 to 100, Fear-avoidance beliefs questionnaire (FABQ) activity from 0 to 24, FABQ work from 0 to 42, Hopkins Symptom Checklist 10 (HSCL-10) from 1 to 4, EuroQol five-dimensional questionnaire (EQ-5D5L) from 0 to 1.

We found that a higher proportion of non-Norwegian was recommended follow-up in primary health care. We found no significant difference in the proportion of Norwegian and non-Norwegian patients receiving individual follow-up or multidisciplinary treatment in the specialist health care system, however, a lower proportion of non-Norwegians were referred to surgery (Table 3).

**Table 3:** Comparison of multidisciplinary treatment in Norwegians and non-Norwegians with odds ratio and confidence interval from Fisher’s exact test.

	Norwegian (%) (n = 8431)	Non-Norwegian (%) (n = 1328)	Odds ratio (95% CI)
Follow-up primary health care	43.4	49.3	1.26 (1.12–1.43)
Follow-up specialist health care			
Individual follow-up	22.9	25.4	1.14 (0.99–1.31)
Multidisciplinary treatment	28.3	28.8	1.02 (0.90–1.16)
Individual	18.6	20.6	1.14 (0.98–1.31)
Group based	14.2	11.3	0.78 (0.65–0.93)
Referred to surgery	5.2	3.2	0.61 (0.43–0.84)

The findings were confirmed with logistic regression analysis (Table 4), where we found that female gender, younger patients, patients with higher education and fear avoidance belief were significantly more likely to receive multidisciplinary follow up. The linearity between the continuous variables and the logit of the outcome was checked. In addition, no influential extreme values were detected and there was no serious multicollinearity between the predictors in the regression analyses.

**Table 4:** Logistic regression analysis with odds ratio for multidisciplinary treatment and 95% confidence interval (CI).

	Odds ratio (95% CI)	P-value
Non-Norwegian vs. Norwegian	0.92 (0.79, 1.06)	0.25
Female vs. male	1.24 (1.12, 1.38)	< 0.001
Age	0.98 (0.98, 0.99)	< 0.001
Vocational studies	1.03 (0.90, 1.17)	0.71
Higher education	1.16 (1.02, 1.32)	0.03
Working vs. non-working	1.10 (0.98, 1.23)	0.09
ODI	1.00 (0.99, 1.00)	0.11
FABQ	1.01 (1.01, 1.02)	< 0.001
NRS activity	1.00 (0.97, 1.03)	0.99
EQ5D5L	1.11 (0.80, 1.56)	0.53

For gender, male serves as reference group. For education, primary and secondary school serves as reference group for both vocational studies and higher education. The reference group for working is non-working participants. Numeric rating scale (NRS). Oswestry Disability Index (ODI). Fear avoidance beliefs questionnaire (FABQ). Hopkins Symptom Checklist 10 (HSCL-10). EuroQol five-dimensional questionnaire (EQ-5D5L).

When exploring people in need of translator, we found that the proportion of patients with need of translator received similar proportion of individual follow-up in the specialist health care system, except that a lower percentage received multidisciplinary treatment compared to people not in need of translator service (Table 5).

**Table 5:** Comparison of multidisciplinary treatment of patients with and without translator.

	No translator (%) (n = 9911)	Translator (%) (n = 149)	Odds ratio (95% CI)
Follow-up primary health care	44%	68%	2.68 (1.88–3.87)
Follow-up specialist health care			
Individual follow-up	23.2	28.2	1.30 (0.89–1.89)
Multidisciplinary treatment	28.6	14.8	0.41 (0.25–0.66)
Individual	18.8	12.8	0.63 (0.37–1.03)
Group based	13.8	2.0	0.12 (0.03–0.38)
Referred to surgery	5.0	1.3	0.26 (0.03–0.95)

## Discussion

This study is the first to describe and compare the symptom burden and multidisciplinary treatment of Norwegian and non-Norwegians in the specialized neck and back units in Norway, using the Norwegian Neck and Back Registry. We found that non-Norwegian patients including those requiring a translator experience a higher symptom burden compared to Norwegian patients, within the Norwegian specialist care system. The proportion of non-Norwegian patients receiving multidisciplinary treatment was similar to that of Norwegian patients, however, fewer non-Norwegians were referred to surgery. However, we observed a lower proportion of patients needing a translator receiving multidisciplinary compared to those who do not require translation service.

Non-Norwegians exhibited reduced back function, increased fear avoidance, and lower health related quality of life in comparison to their Norwegian counterparts. These findings align with previous studies conducted in Denmark, where immigrants reported higher levels of daily pain compared to ethnic Danes. However, cultural variance in these factors remains poorly investigated^29^. In Norway, the aim is that 30% of the patients receive a multidisciplinary follow-up. In the current study, the proportion of patients receiving this type of treatment is approximately 30% for all patient groups, except for those requiring a translator. Therefore, Norwegian Physical Medicine and Rehabilitation clinics should consider implementing a change in strategy to increase the proportion of patients needing a translator who receive multidisciplinary treatment. It may even be necessary to prioritize higher levels of multidisciplinary treatment for non-Norwegians and individuals requiring a translator, given their higher symptom burden.

When treating patients with back and neck pain, it is recommended to adopt a holistic approach that recognizes the dynamic relationship between biological, psychological, and social factors influencing the pain experience^30^. Treatment traditions and guidelines in Western Europe, including Norway, are based on the biopsychosocial model. These approaches combine activity advice, exercises, and cognitive interventions in accordance with international recommendations^12,31^. In the Norwegian Specialist care system, the treatment of patients with neck and back pain typically involves a multidisciplinary approach that includes physicians, physiotherapists, psychologists, occupational therapists, and social workers. These interventions are complex and require active patient participation. However, Norwegian level and cultural differences can create barriers to participation. Furthermore, in Norway, most multidisciplinary treatments for back and neck pain are done in group settings with Norwegian as the language. Previous randomized controlled trials have often excluded patients requiring a translator, creating a gap in our understanding of the best treatment for these individuals. Future studies should investigate the effectiveness of both individual and group interventions that involve translators or employ other methods such as educational technology in different languages to overcome barriers to multidisciplinary treatment for patients who require translation services.

The importance of developing treatment options for those requiring translation services is emphasized by the higher percentage of such individuals being directed back to follow-up in primary health care. Despite this, many patients referred to specialist health care were already receiving treatment in primary care before the referral. While our study could not investigate the follow-up of these recommendations, primary health care follow-up appears more disjointed and less interdisciplinary than specialist health care. Moreover, accessing translators may be simpler in specialist health care.

Social inequality in health services and the underrepresentation of immigrant groups in research are well-known issues. There is a significant knowledge gap regarding how our health services can effectively address the healthcare needs and expectations of different cultural groups. Although the funding of the Norwegian healthcare system is public and aims to provide equal access to healthcare services. A previous study^32^ revealed that Norwegian immigrants with musculoskeletal disorders (MSD) express lower satisfaction with health services compared to their Norwegian counterparts, indicating a lack of equality in the treatment of immigrants and other minority groups with MSD. A qualitative study among Polish workers^33^ in Norway revealed that language difficulties and lack of knowledge about the Norwegian healthcare system created communication barriers, hindering access to healthcare. On the other hand, the presence of Polish networks and the perception of equal treatment facilitated access to healthcare services. Another possible cause of this dissatisfaction is a significant gap between treatment expectations and the actual type of treatment provided^34^. Studies^35,36^ from other countries have indicated that expectations based on experiences from their home country, lack of culturally sensitive healthcare services, and negative experiences with these services in the new country can all influence access to healthcare. However, it is essential to consider migrants' utilization of healthcare services in the context of the inequalities in health that exist in Norway and globally^37^.

In musculoskeletal research^10,38,39^, there is typically a low representation of immigrants in clinical trials. This raises questions about the external validity and direct applicability of randomized trial results in clinical practice^10^. Future studies should investigate social and ethnic disparities in treatment expectations and needs, as well as reasons for service provision.

Previous studies^37^ indicates that socioeconomic status among immigrants has an impact on health, where both mental and physical health is better among those with higher education, are employed, and have good economy. A Belgian study^40^ found that Turkish immigrants’ emotional experiences become more similar to those of Belgians the longer they live in Belgium. Research^41–43^ have found that poor language skills and lower acculturation are associated with poor health, probably due to difficulties in understanding health information, utilizing healthcare services, and receiving high-quality care. Conversely, health issues can be a barrier towards mastering the host language^9^.

Pain is a physical and emotional symptom at the same time. Most of the outcomes in our study are somehow related to emotions. Emotions are not universal, but related to concepts that have diverse meanings in different cultures^44^. Even for properly translated questionnaires the meanings might differ^40,41^. Each patient's symptom burden, including emotional aspects, is unique. In comparing means at the group level we miss some of the uniqueness that describe every single patient. Still our study reveals that the symptoms are best interpreted as biopsychosocial. Immigrants experience more intense symptoms than Norwegians. This calls for proactive measures to promote a better understanding and treatment of immigrants with back and neck pain. This includes both translation and equality in individual and group based multidisciplinary treatment.

Aligning with the Norwegian authorities' emphasis on equal rights, the present findings highlight possible unmet treatment needs among non-Norwegians, particularly patients requiring translation services. This includes addressing cultural differences, language barriers, and understanding the specific healthcare needs of immigrant communities. Efforts should be made to address these barriers and provide culturally sensitive and language-appropriate healthcare services to all inhabitants, ensuring their equitable access to quality care. In Norway, patients have the right to access a translator when needed; however, there is currently no research evaluating the need for and utilization of translators in the Norwegian healthcare system.

Our findings revealed that multidisciplinary treatment was more commonly received by younger patients, females, those with higher education, and individuals exhibiting fear avoidance belief. Females have a higher health care seeking ratio than men, which also may influence the preferences for multidisciplinary follow up found in this study^45^. In a randomized trial^46^ conducted in Norway; it was observed that a reduction in fear avoidance belief following treatment is a significant predictor for successful return to work. Thus, the treatment modalities in Norway tends to focus on patient functioning and belief.

### Methodological considerations

One of the strengths of this study is its large study population, which included over 10,000 patients. The inclusion of 71% of all patients referred to the participating outpatient clinics is considered acceptable, although we cannot exclude a potential selection bias due to the requirement of filling out the questionnaire in either Norwegian or English. Another strength is the register-based approach, as it seeks to include all patients, increasing the heterogeneity of the population studied. This means that a considerable proportion of the remaining 29% may consist of non-Norwegians and patients requiring a translator, who may not possess adequate skills in Norwegian or English. Another limitation is the registration of nationality, which does not capture information about language skills, cultural background, or ethnicity. Therefore, we suggest that the NNRR implements additional questions to explore these factors.

We found that fewer non-Norwegians were referred to surgery, compared to Norwegians. However, a limitation of this study is that we do not have any information to explain this difference. It might be due to differences in comorbidity or personal preferences, however, this warrants further follow-up of unmet needs among non-Norwegians with back and neck pain.

Another possible way forward is the translation of PROMS in the NNRR to additional languages. In a recent study, Alpers et al.^47^ discovered that many immigrant patients encounter difficulties when filling out patient-reported outcome measures (PROMs) or questionnaires, even when they are professionally translated into their native language. Language difficulties may contribute to the heightened symptom burden observed among non-Norwegians and patients requiring translation services, as the latter probably have had help filling out the PROMs.

## Conclusion

In conclusion, we found that non-Norwegian patients experience a higher symptom burden compared to Norwegians patients, within the Norwegian specialist care system. In addition, we observed a lower proportion of patients needing a translator receiving multidisciplinary compared to those who do not require translation service. Thus, future studies should assess the effectiveness of both individual and group-based interventions that utilize translators or implement other strategies to reduce barriers to multidisciplinary treatment.

## Data Availability

The data that support the findings of this study are available from the Norwegian neck and back registry, but restrictions apply to the availability of these data, which were used under license for the current study, and so are not publicly available. Data are however available from the authors upon reasonable request and with permission of the Norwegian neck and back registry.

## Abbreviations

EQ-5D-5L: EuroQol five-dimensional questionnaire
FABQ: Fear avoidance belief questionnaire
HSCL-10: Hopkins symptom checklist 10
MSD: Musculoskeletal disorders
NDI: Neck disability index
NNRR: Norwegian neck and back register
NRS: Numeric rating scale
ODI: Oswestry Disability Index
PROM: Patient reported outcome measure
SHC: Subjective health complain
